# Supplementary material for: Pyruvate Dehydrogenase Kinase 1 inhibition mediated oxidative phosphorylation enhancement in cartilage promotes osteoarthritis progression
Source: BMC Musculoskelet Disord. 2023 Jul 20;24:597. doi: 10.1186/s12891-023-06585-6 (PMC10357736; doi:10.1186/s12891-023-06585-6)
Supplement: Supplementary file 1 — Additional file 1. [file 12891_2023_6585_MOESM1_ESM.zip › KEGG.Gsea.1653622432464/KEGG_OLFACTORY_TRANSDUCTION.html]

Details for gene set KEGG\_OLFACTORY\_TRANSDUCTION[GSEA]

|  || Dataset | 55457\_collapsed\_to\_symbols.cls.cls #OA\_versus\_normal.cls.cls #OA\_versus\_normal\_repos |
| Phenotype | cls.cls#OA\_versus\_normal\_repos |
| Upregulated in class | normal |
| GeneSet | KEGG\_OLFACTORY\_TRANSDUCTION |
| Enrichment Score (ES) | -0.2681351 |
| Normalized Enrichment Score (NES) | -1.093372 |
| Nominal p-value | 0.28525123 |
| FDR q-value | 0.53316516 |
| FWER p-Value | 1.0 |
Table: GSEA Results Summary

  

Fig 1: Enrichment plot: KEGG\_OLFACTORY\_TRANSDUCTION      
 Profile of the Running ES Score & Positions of GeneSet Members on the Rank Ordered List

  

| SYMBOL | TITLE | RANK IN GENE LIST | RANK METRIC SCORE | RUNNING ES | CORE ENRICHMENT || 1 | GUCA1A | guanylate cyclase activator 1A [Source:HGNC Symbol;Acc:HGNC:4678] | 316 | 0.739 | 0.0152 | No |
| 2 | PRKACB | protein kinase cAMP-activated catalytic subunit beta [Source:HGNC Symbol;Acc:HGNC:9381] | 771 | 0.576 | 0.0109 | No |
| 3 | CAMK2A | calcium/calmodulin dependent protein kinase II alpha [Source:HGNC Symbol;Acc:HGNC:1460] | 916 | 0.538 | 0.0287 | No |
| 4 | CALM3 | calmodulin 3 [Source:HGNC Symbol;Acc:HGNC:1449] | 1039 | 0.510 | 0.0467 | No |
| 5 | CALML5 | calmodulin like 5 [Source:HGNC Symbol;Acc:HGNC:18180] | 1261 | 0.465 | 0.0546 | No |
| 6 | OR2J2 | olfactory receptor family 2 subfamily J member 2 [Source:HGNC Symbol;Acc:HGNC:8260] | 1367 | 0.448 | 0.0706 | No |
| 7 | CLCA2 | chloride channel accessory 2 [Source:HGNC Symbol;Acc:HGNC:2016] | 1787 | 0.386 | 0.0587 | No |
| 8 | OR7A5 | olfactory receptor family 7 subfamily A member 5 [Source:HGNC Symbol;Acc:HGNC:8368] | 1943 | 0.368 | 0.0665 | No |
| 9 | CLCA4 | chloride channel accessory 4 [Source:HGNC Symbol;Acc:HGNC:2018] | 1967 | 0.364 | 0.0843 | No |
| 10 | GUCY2D | "guanylate cyclase 2D, retinal [Source:HGNC Symbol;Acc:HGNC:4689]" | 2832 | 0.267 | 0.0314 | No |
| 11 | CAMK2G | calcium/calmodulin dependent protein kinase II gamma [Source:HGNC Symbol;Acc:HGNC:1463] | 2900 | 0.261 | 0.0403 | No |
| 12 | OR11A1 | olfactory receptor family 11 subfamily A member 1 [Source:HGNC Symbol;Acc:HGNC:8176] | 3121 | 0.238 | 0.0360 | No |
| 13 | OR12D2 | olfactory receptor family 12 subfamily D member 2 [Source:HGNC Symbol;Acc:HGNC:8178] | 3191 | 0.232 | 0.0431 | No |
| 14 | OR2B2 | olfactory receptor family 2 subfamily B member 2 [Source:HGNC Symbol;Acc:HGNC:13966] | 3815 | 0.175 | 0.0040 | No |
| 15 | OR1D5 | olfactory receptor family 1 subfamily D member 5 [Source:HGNC Symbol;Acc:HGNC:8186] | 4113 | 0.145 | -0.0114 | No |
| 16 | OR10H1 | olfactory receptor family 10 subfamily H member 1 [Source:HGNC Symbol;Acc:HGNC:8172] | 4167 | 0.141 | -0.0079 | No |
| 17 | GRK3 | G protein-coupled receptor kinase 3 [Source:HGNC Symbol;Acc:HGNC:290] | 4208 | 0.137 | -0.0036 | No |
| 18 | OR1A2 | olfactory receptor family 1 subfamily A member 2 [Source:HGNC Symbol;Acc:HGNC:8180] | 4314 | 0.127 | -0.0049 | No |
| 19 | CNGB1 | cyclic nucleotide gated channel subunit beta 1 [Source:HGNC Symbol;Acc:HGNC:2151] | 4509 | 0.112 | -0.0140 | No |
| 20 | OR52A1 | olfactory receptor family 52 subfamily A member 1 [Source:HGNC Symbol;Acc:HGNC:8318] | 4655 | 0.100 | -0.0199 | No |
| 21 | OR2S2 | olfactory receptor family 2 subfamily S member 2 [Source:HGNC Symbol;Acc:HGNC:8276] | 5152 | 0.059 | -0.0553 | No |
| 22 | GNAL | G protein subunit alpha L [Source:HGNC Symbol;Acc:HGNC:4388] | 5158 | 0.059 | -0.0526 | No |
| 23 | OR12D3 | olfactory receptor family 12 subfamily D member 3 [Source:HGNC Symbol;Acc:HGNC:13963] | 5159 | 0.059 | -0.0494 | No |
| 24 | OR10J1 | olfactory receptor family 10 subfamily J member 1 [Source:HGNC Symbol;Acc:HGNC:8175] | 5255 | 0.052 | -0.0540 | No |
| 25 | CLCA1 | chloride channel accessory 1 [Source:HGNC Symbol;Acc:HGNC:2015] | 5363 | 0.043 | -0.0600 | No |
| 26 | OR3A2 | olfactory receptor family 3 subfamily A member 2 [Source:HGNC Symbol;Acc:HGNC:8283] | 6097 | -0.012 | -0.1165 | No |
| 27 | CNGA3 | cyclic nucleotide gated channel subunit alpha 3 [Source:HGNC Symbol;Acc:HGNC:2150] | 6163 | -0.017 | -0.1206 | No |
| 28 | OR10H3 | olfactory receptor family 10 subfamily H member 3 [Source:HGNC Symbol;Acc:HGNC:8174] | 6287 | -0.026 | -0.1288 | No |
| 29 | CALML3 | calmodulin like 3 [Source:HGNC Symbol;Acc:HGNC:1452] | 6348 | -0.032 | -0.1317 | No |
| 30 | OR1A1 | olfactory receptor family 1 subfamily A member 1 [Source:HGNC Symbol;Acc:HGNC:8179] | 6444 | -0.038 | -0.1371 | No |
| 31 | OR2B6 | olfactory receptor family 2 subfamily B member 6 [Source:HGNC Symbol;Acc:HGNC:8241] | 6524 | -0.044 | -0.1409 | No |
| 32 | OR1D2 | olfactory receptor family 1 subfamily D member 2 [Source:HGNC Symbol;Acc:HGNC:8183] | 6577 | -0.048 | -0.1423 | No |
| 33 | PDE1C | phosphodiesterase 1C [Source:HGNC Symbol;Acc:HGNC:8776] | 6819 | -0.064 | -0.1576 | No |
| 34 | OR51E2 | olfactory receptor family 51 subfamily E member 2 [Source:HGNC Symbol;Acc:HGNC:15195] | 6898 | -0.072 | -0.1598 | No |
| 35 | ARRB2 | arrestin beta 2 [Source:HGNC Symbol;Acc:HGNC:712] | 7255 | -0.100 | -0.1822 | No |
| 36 | OR1G1 | olfactory receptor family 1 subfamily G member 1 [Source:HGNC Symbol;Acc:HGNC:8204] | 7387 | -0.109 | -0.1865 | No |
| 37 | OR1F1 | olfactory receptor family 1 subfamily F member 1 [Source:HGNC Symbol;Acc:HGNC:8194] | 7409 | -0.111 | -0.1821 | No |
| 38 | OR7C2 | olfactory receptor family 7 subfamily C member 2 [Source:HGNC Symbol;Acc:HGNC:8374] | 7443 | -0.114 | -0.1785 | No |
| 39 | PDC | phosducin [Source:HGNC Symbol;Acc:HGNC:8759] | 7820 | -0.143 | -0.2001 | No |
| 40 | ADCY3 | adenylate cyclase 3 [Source:HGNC Symbol;Acc:HGNC:234] | 8572 | -0.199 | -0.2479 | No |
| 41 | OR2W1 | olfactory receptor family 2 subfamily W member 1 [Source:HGNC Symbol;Acc:HGNC:8281] | 8780 | -0.215 | -0.2524 | Yes |
| 42 | CAMK2B | calcium/calmodulin dependent protein kinase II beta [Source:HGNC Symbol;Acc:HGNC:1461] | 8782 | -0.215 | -0.2409 | Yes |
| 43 | OR2H1 | olfactory receptor family 2 subfamily H member 1 [Source:HGNC Symbol;Acc:HGNC:8252] | 8851 | -0.221 | -0.2343 | Yes |
| 44 | OR2H2 | olfactory receptor family 2 subfamily H member 2 [Source:HGNC Symbol;Acc:HGNC:8253] | 9097 | -0.239 | -0.2405 | Yes |
| 45 | PRKG2 | protein kinase cGMP-dependent 2 [Source:HGNC Symbol;Acc:HGNC:9416] | 9126 | -0.242 | -0.2296 | Yes |
| 46 | OR2F2 | olfactory receptor family 2 subfamily F member 2 [Source:HGNC Symbol;Acc:HGNC:8247] | 9316 | -0.257 | -0.2305 | Yes |
| 47 | OR1E1 | olfactory receptor family 1 subfamily E member 1 [Source:HGNC Symbol;Acc:HGNC:8189] | 9744 | -0.296 | -0.2478 | Yes |
| 48 | OR10H2 | olfactory receptor family 10 subfamily H member 2 [Source:HGNC Symbol;Acc:HGNC:8173] | 10006 | -0.322 | -0.2508 | Yes |
| 49 | CALM1 | calmodulin 1 [Source:HGNC Symbol;Acc:HGNC:1442] | 10136 | -0.334 | -0.2428 | Yes |
| 50 | OR2F1 | olfactory receptor family 2 subfamily F member 1 [Source:HGNC Symbol;Acc:HGNC:8246] | 10148 | -0.336 | -0.2255 | Yes |
| 51 | PRKACG | protein kinase cAMP-activated catalytic subunit gamma [Source:HGNC Symbol;Acc:HGNC:9382] | 10466 | -0.370 | -0.2303 | Yes |
| 52 | CALM2 | calmodulin 2 [Source:HGNC Symbol;Acc:HGNC:1445] | 10579 | -0.382 | -0.2184 | Yes |
| 53 | PRKACA | protein kinase cAMP-activated catalytic subunit alpha [Source:HGNC Symbol;Acc:HGNC:9380] | 10611 | -0.384 | -0.2001 | Yes |
| 54 | OR5I1 | olfactory receptor family 5 subfamily I member 1 [Source:HGNC Symbol;Acc:HGNC:8347] | 10616 | -0.385 | -0.1796 | Yes |
| 55 | OR7A17 | olfactory receptor family 7 subfamily A member 17 [Source:HGNC Symbol;Acc:HGNC:8363] | 10697 | -0.392 | -0.1647 | Yes |
| 56 | PRKG1 | protein kinase cGMP-dependent 1 [Source:HGNC Symbol;Acc:HGNC:9414] | 10776 | -0.403 | -0.1491 | Yes |
| 57 | OR2J3 | olfactory receptor family 2 subfamily J member 3 [Source:HGNC Symbol;Acc:HGNC:8261] | 10789 | -0.404 | -0.1282 | Yes |
| 58 | OR7C1 | olfactory receptor family 7 subfamily C member 1 [Source:HGNC Symbol;Acc:HGNC:8373] | 10945 | -0.424 | -0.1174 | Yes |
| 59 | OR7A10 | olfactory receptor family 7 subfamily A member 10 [Source:HGNC Symbol;Acc:HGNC:8356] | 11448 | -0.496 | -0.1298 | Yes |
| 60 | OR3A1 | olfactory receptor family 3 subfamily A member 1 [Source:HGNC Symbol;Acc:HGNC:8282] | 11554 | -0.508 | -0.1106 | Yes |
| 61 | OR3A3 | olfactory receptor family 3 subfamily A member 3 [Source:HGNC Symbol;Acc:HGNC:8284] | 11603 | -0.516 | -0.0864 | Yes |
| 62 | PRKX | protein kinase X-linked [Source:HGNC Symbol;Acc:HGNC:9441] | 11634 | -0.522 | -0.0606 | Yes |
| 63 | GUCA1B | guanylate cyclase activator 1B [Source:HGNC Symbol;Acc:HGNC:4679] | 12002 | -0.593 | -0.0572 | Yes |
| 64 | OR2C1 | olfactory receptor family 2 subfamily C member 1 [Source:HGNC Symbol;Acc:HGNC:8242] | 12366 | -0.696 | -0.0479 | Yes |
| 65 | OR7E24 | olfactory receptor family 7 subfamily E member 24 [Source:HGNC Symbol;Acc:HGNC:8396] | 12451 | -0.725 | -0.0154 | Yes |
| 66 | OR10C1 | olfactory receptor family 10 subfamily C member 1 [Source:HGNC Symbol;Acc:HGNC:8165] | 12776 | -0.932 | 0.0097 | Yes |
Table: GSEA details [plain text format]

  

Fig 2: KEGG\_OLFACTORY\_TRANSDUCTION      
 Blue-Pink O' Gram in the Space of the Analyzed GeneSet

  

Fig 3: KEGG\_OLFACTORY\_TRANSDUCTION: Random ES distribution      
 Gene set null distribution of ES for **KEGG\_OLFACTORY\_TRANSDUCTION**
